# GLS2 reduces the occurrence of epilepsy by affecting mitophagy function in mouse hippocampal neurons

**DOI:** 10.1111/cns.70036

**Published:** 2024-10-15

**Authors:** Yuan Gao, Limin Ma, Jinxian Yuan, Yunyi Huang, Yuenan Ban, Peng Zhang, Dandan Tan, Minxue Liang, Zhipeng Li, Chen Gong, Tao Xu, Xiaolan Yang, Yangmei Chen

**Affiliations:** ^1^ Department of Neurology The Second Affiliated Hospital of Chongqing Medical University Chongqing China; ^2^ Department of Gerontology Chongqing University Three Gorges Hospital Chongqing China; ^3^ Department of Neurology Chongqing Medical University Affiliated Second Hospital Affiliated Fengjie Hospital Chongqing China

**Keywords:** epilepsy, GLS2, mitophagy, neuron, reactive oxygen species (ROS), seizure

## Abstract

**Background:**

Altered mitophagy has been observed in various neurological disorders, such as epilepsy. The role of mitophagy in causing neuronal damage during epileptic episodes is significant, and recent research has indicated that GLS2 plays a crucial role in regulating autophagy. However, exactly how GLS2 affects epilepsy is still unclear.

**Aims:**

To investigate the expression and distribution characteristics of GLS2 in epilepsy, and then observed the changes in behavior and electrophysiology caused by overexpression of GLS2 in epileptic mice, and determined whether GLS2 regulated seizure‐like changes in the mouse model through the protective mechanism of mitophagy.

**Results:**

The expression of GLS2 in a kainic acid (KA)‐induced epileptic mouse model and aglutamate‐inducedneuronal excitatory damage in HT22 cells model was downregulation. In brief, overexpression of GLS2 can alleviate epileptic activity. Subsequently, we demonstrated that GLS2 interacts with mitophagy‐related proteins in a KA‐induced epilepsy mouse model. Mechanistically, overexpression of GLS2 inhibited mitophagy in epileptic mice, downregulating the expression of LC3 and reducing ROS production.

**Conclusions:**

This study proves the GLS2 expression pattern is abnormal in epileptic mice. The function of mitophagy in hippocampal neurons is affected by GLS2, and overexpression of GLS2 can reduce the occurrence of seizure‐like events (SLEs) by altering mitophagy function. Thus, GLS2 might control seizures, and our findings provide a fresh avenue for antiepileptic treatment and offer novel insights into treating and preventing epilepsy.

## Graphical abstract

The mechanism diagram of mitophagy in epileptic discharge neurons. Overexpression of GLS2 protected the mitochondrial inner membrane from damage and inhibited mitochondrial autophagy in hippocampal neurons with epileptic discharge, thereby reducing the generation of ROS.

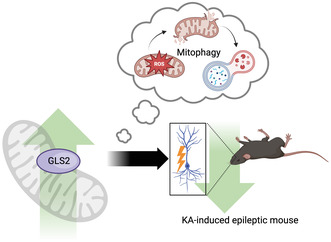

## 1 INTRODUCTION

With a complicated etiology and pathogenesis, epilepsy is a heterogeneous disease. Although much research has been conducted on this topic, the fundamental causes and underlying mechanisms of epilepsy have not been identified.^1^, ^2^ Epilepsy is a common neurological disease that affects many patients and their families to a certain extent, and further research is needed to help develop new methods for treating epileptic seizures in patients with epilepsy.^3^, ^4^

Mitochondrial dysfunction can generate reactive oxygen species (ROS), injuring cellular proteins and DNA or triggering programmed cell death.^5^ In response, eukaryotic cells have created systems that use macroautophagy to sequester and destroy mitochondria.^6^, ^7^ An imbalance in mitophagy is one of the causes of abnormal neuronal discharge and epileptic seizures, and understanding the mechanism involved is urgent for curing epilepsy patients.^8^, ^9^

Glutaminase 2 (GLS2) is a mitochondrial phosphate‐activated glutaminase (GLS) that catalyzes the hydrolysis of glutamine to stoichiometric quantities of glutamate and ammonium.^10^, ^11^ GLS2 was first discovered in the liver and kidneys and is closely related to cancer cell proliferation.^12^, ^13^, ^14^ In recent years, increasing evidence has shown that it is highly expressed in the brain.^15^, ^16^, ^17^ GLS2 can regulate the generation of ROS,^18^, ^19^ and its expression is speculated to be inseparable from mitophagy function.^20^, ^21^

As a result, the goal of this study was to investigate the expression patterns of GLS2 in epilepsy and to determine whether GLS2 regulates epileptic‐like alterations in kainic acid (KA)‐induced epilepsy mouse models via the protective mechanism of mitophagy. Furthermore, we investigated the possible mechanism through which GLS2‐induced mitophagy modulates epileptic behavior.

## 2 RESULTS

### 2.1 The expression pattern of GLS2 in epilepsy

**FIGURE 1:**
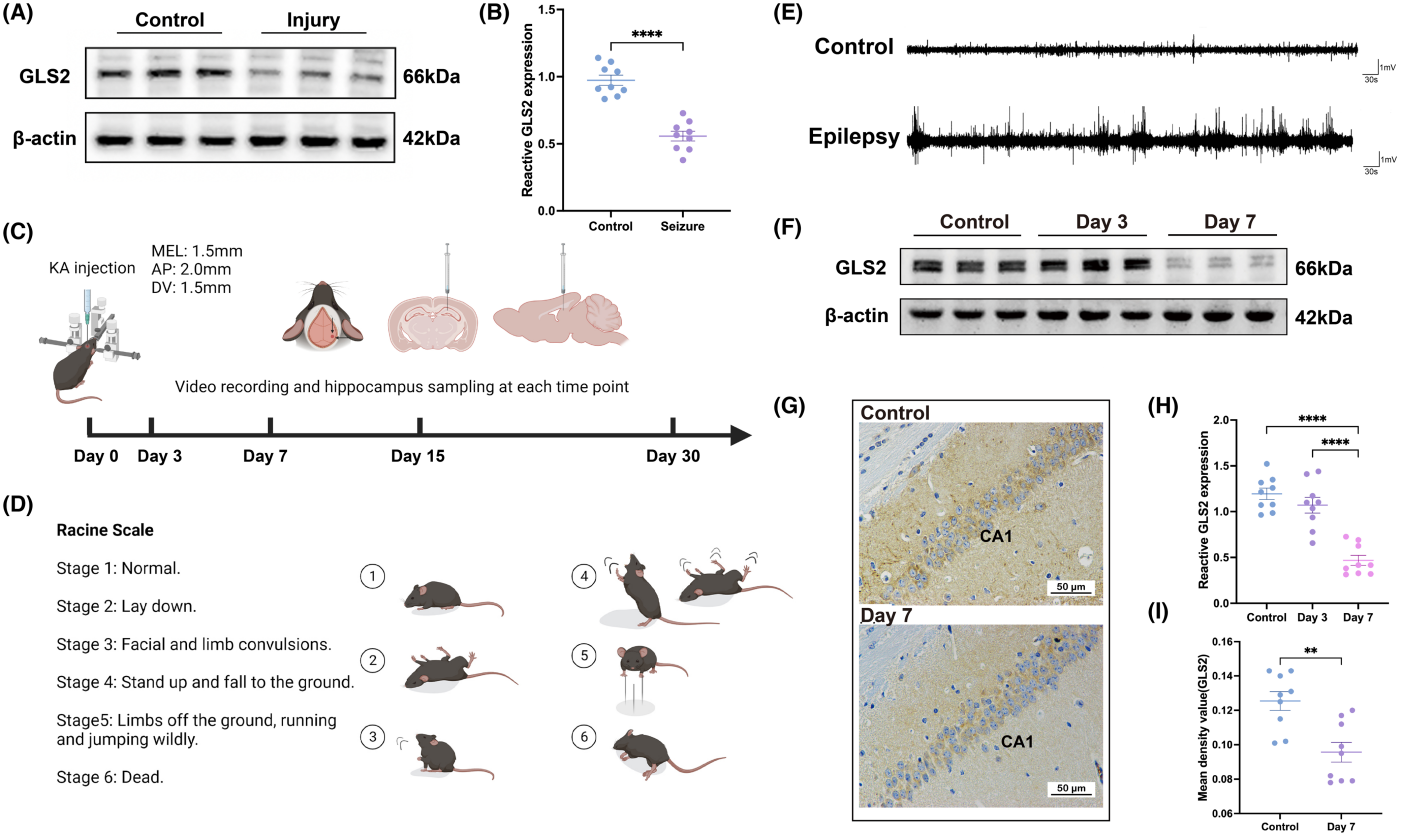
(A) The expression levels of GLS2 in HT22 cells were evaluated by Western blot following glutamate treatment, and the statistical analyses (B) were also conducted (****p < 0.0001, Student's *t*‐test with *n* = 9 per group). (C) Time points for modeling and sampling of KA‐induced epileptic mice. (D) Racine scale. (E) Representative image of KA‐induced epileptiform discharges. (F) Western blot images of hippocampal GLS2 expression in the control group; at three (Day 3) and seven (Day 7) days after modeling; and (H) the associated statistical analysis (****P < 0.0001; one‐way ANOVA with n = 9 per group). (G) Representative image of GLS2 expression in the mouse hippocampal CA1 region was measured by immunohistochemistry, and (I) the corresponding statistical analyses were performed (**P < 0.01; Student’s t‐test with n = 9 per group).

To understand the role of GLS2 in the glutamate‐induced neuronal excitatory injury model, We first used glutamate to induce excitatory damage in HT22 cells to simulate neuronal damage after epilepsy (Injury group). Western blot analysis and statistical analysis revealed that, compared with the control group, the GLS2 expression in the Seizure group was substantially lower. (Figure 1A,B). A KA‐induced epilepsy model was developed to explore the connection between GLS2 and epilepsy more thoroughly. Behavioral changes in the mice were detected via real‐time video, and the occurrence of epileptic events was evaluated by using the Racine scale (Figure 1D) and hippocampal local field potential (LFP) recordings (Figure 1E) and mice that successfully constructed the model were selected for the following process. After successful modeling, the hippocampus was dissected for Western blot analysis on the 3rd and 7th, days. Western blot (Figure 1H) and immunohistochemistry (Figure 1G), and corresponding statistical analysis (Figure 1I) showed a decrease in GLS2 expression on the seventh day after modeling.

### 2.2 The localization of GLS2 in the hippocampus

**FIGURE 2:**
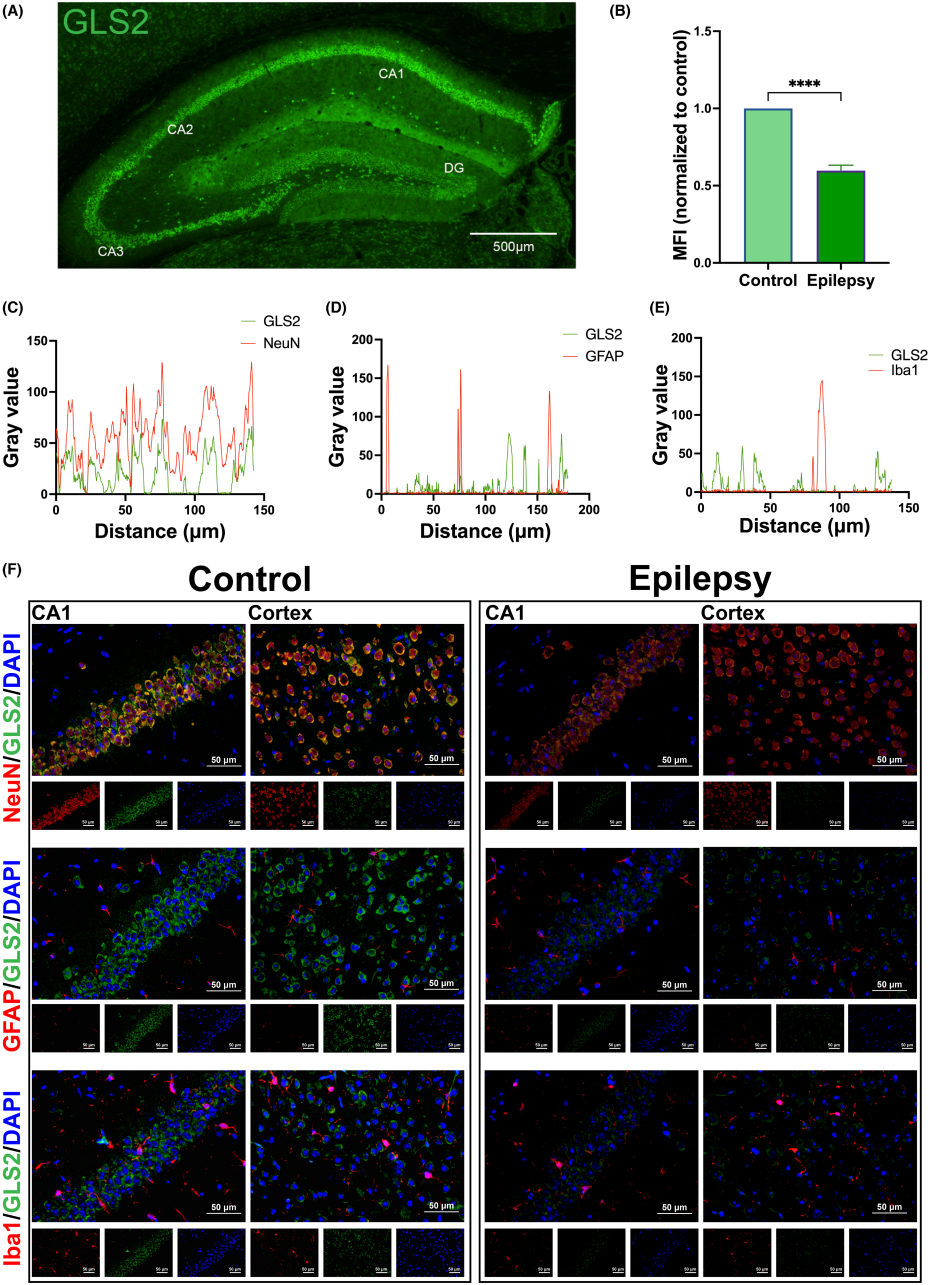
(A) Typical GLS2 expression immunofluorescence images of mouse hippocampal tissue. (the scale bar =500 μm). (B) Corresponding statistical analyses of the MFI of GLS2 (****p < 0.0001; Student's *t* test with *n* = 9 per group). (F) Representative images of GLS2 (green) colocalization with NeuN (C), GFAP (D), and Iba1 (E) (red) via dual‐label immunofluorescence (the scale bar = 50 μm).

In the layer of granular cell and pyramidal neurons of the hippocampus, immunofluorescent GLS2 staining (green staining) revealed extensive dispersion (Figure 2A). Subsequently, we conducted immunofluorescence experiments on the hippocampus of mice 7 days after KA injection. The results of the immunofluorescence experiment indicate that GLS2 was colocalized with the neuronal markers NeuN and not with the astrocytic markers GFAP and microglial markers Iba1. (Figure 2C–F). Stronger GLS2 immunofluorescence was found in the CA1 region of the epileptic group than the control group based on subsequent analysis of the mean fluorescence intensity (MFI; Figure 2B).

### 2.3 Overexpression of GLS2 suppresses seizure susceptibility in vivo

**FIGURE 3:**
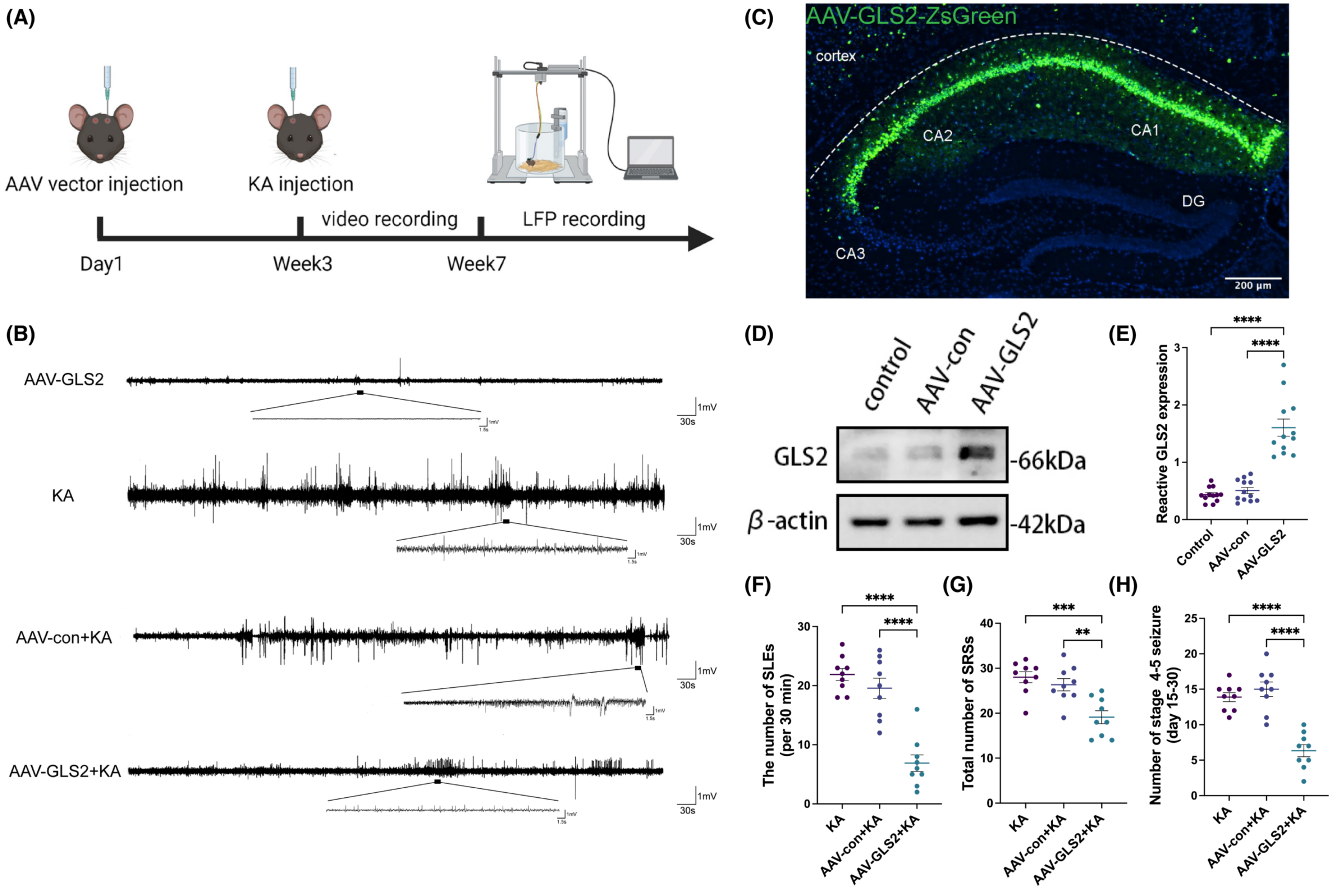
GLS2 expression was induced in vivo via the AAV vector. (A) Schematic illustration of the trial setup. (B) A comparison of representative images derived from hippocampal LFP recordings with the associated statistical analysis (F) of the number of SLEs identified from those recordings per 30 min (****p < 0.0001, one‐way ANOVA with *n* = 9 per group). (C) Fluorescence images of the distribution of ZsGreen (the scale bar = 200 μm). (D) Western blotting images for GLS2 expression and (E) the corresponding statistical analyses (****p < 0.0001; one‐way ANOVA with *n* = 9 per group). (G) Statistical analysis of the number of SRSs (**p < 0.01, ***p < 0.001; one‐way ANOVA with *n* = 9 per group). (H) Statistical analysis of the number of Stage 4–5 seizures on the 15th to 30th days after modeling (**p < 0.01, ***p < 0.001; one‐way ANOVA with n = 9 per group).

Three weeks before the establishment of the KA model, the AAV virus vector was injected into the CA1 region of the bilateral hippocampus of mice to induce overexpression of GLS2 (Figure 3A). AAV vector‐mediated GLS2 expression was validated by Western blot and immunofluorescence assays (Figure 3C,D). The AAV‐GLS2 group presented a significant increase in GLS2 expression compared to that in the AAV‐con and control groups. Moreover, there was no discernible difference between the latter two groups, indicating that the AAV vector did not cause interference. The findings are displayed in a statistical graph (Figure 3F; ****p < 0.0001; one‐way ANOVA with *n* = 9 per group). After the satisfactory confirmation of GLS2 overexpression, a model of KA‐induced epilepsy was developed. LFP field potential monitoring of the mouse hippocampal CA1 area was performed on the 30th day after KA injection (Figure 3B). To determine the impact of AAV‐GLS2 on mice, the LFP in the CA1 region of the mouse hippocampus was monitored at the third week after AAV‐GLS2 injection and before KA injection. Every 30 min, the number of seizure‐like episodes (SLEs) with amplitudes greater than 1 mV and durations longer than 5 s in the LFP of the hippocampus in the AAV‐GLS2 group, KA group, AAV‐con+KA group, and AAV‐GLS2 + KA group were counted. SLE was not detected in the AAV‐GLS2 group, according to the LFP recordings (Figure 3B). In contrast, in the AAV‐GLS2 + KA group, SLEs were less frequent and shorter than they were in the KA and AAV‐con+KA groups (Figure 3F; ****p < 0.0001; one‐way ANOVA; *n* = 6 per group). However, the number of SLEs did not change between the AAV‐con+KA and KA groups (*p* = 0.898; Figure 3F). In addition, the number of SRSs and stage 4–5 seizures on the 15th to 30th days after modeling in the AAV‐GLS2 group were less than those in the other groups (**p < 0.01, ***p < 0.001; one‐way ANOVA with *n* = 9 per group).

### 2.4 Overexpression of GLS2 in the formation of epilepsy decreases the role of mitophagy in epilepsy

**FIGURE 4:**
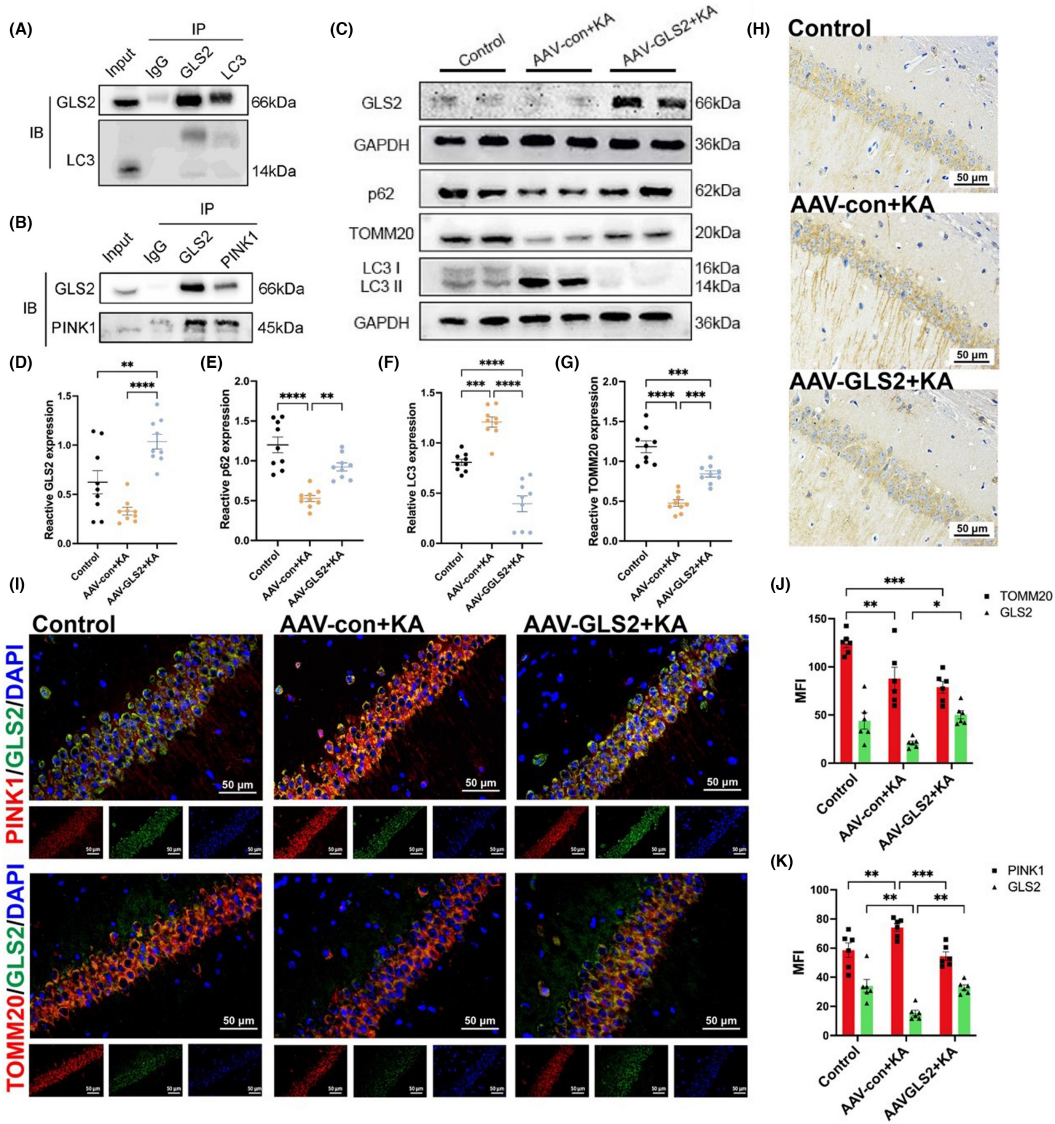
(A) Results of the Co‐IP assay for GLS2 and LC3 in the hippocampus of KA‐induced epileptic mice. (B) Results of Co‐IP assays for GLS2 and PINK1 in the hippocampus of KA‐induced epileptic mice. (C) Representative Western blot images showed that during epilepsy development, the expression of p62 and TOMM20 decreased, while the expression of LC3 II increased. In contrast, overexpression of GLS2 increased the expression of p62, and TOMM20 reduced the expression of LC3 during epilepsy development and (D–G) the corresponding statistical analyses (**p < 0.01, ***p < 0.001, ****p < 0.0001; one‐way ANOVA with *n* = 9 per group). (H) Typical images of LC3‐positive cells stained with immunohistochemistry. The AAV‐con+KA group exhibited robust immunoreactive staining of LC3 in the hippocampal CA1 region, whereas the AAV‐GLS2 + KA group displayed less intense positive staining. This contrasts with the mild staining of the control group (the scale bar = 50 μm). (I) Representative dual‐label immunofluorescence images (the scale bar = 50 μm), with the left indicating colocalization of PINK1 and GLS2 and (J) the statistical analyses of the mean fluorescence values of PINK1 and GLS2 (**p < 0.01, ***p < 0.001; two‐way ANOVA with *n* = 6 per group); the right indicating colocalization of TOMM20 and GLS2; and (K) the statistical analyses of the mean fluorescence values of TOMM20 and GLS2 (*p < 0.05, **p < 0.01, ***p < 0.001; two‐way ANOVA with *n* = 6 per group).

To determine the interaction between hippocampal GLS2 and mitophagy‐related proteins in epileptic mice, the interactions between GLS2 and LC3, as well as between GLS2 and PTEN‐induced kinase 1 (PINK1), were detected via a Co‐IP assay (Figure 4A,B). Based on the Co‐IP results, GLS2 was speculated to be closely related to mitophagy in KA‐induced epileptic mice. Western blot analysis was used to measure the expression levels of the mitophagy indicator microtubule‐associated protein 1 light chain 3 (LC3), selective autophagy receptor SQSTM1/p62, the mitochondrial outer membrane indicator TOMM20 and GLS2 in the control, AAV‐con+KA, and AAV‐GLS2 + KA groups to examine the impact of GLS2 on mitophagy during the development of epilepsy. When autophagy occured, LC3I usually converted to LC3II, accompanied by the degradation of p62. Compared with that in the control group, the total TOMM20 in the hippocampus of the AAV‐con+KA group was downregulated, p62 was downregulate and LC3II was upregulated. The overall TOMM20 level in the hippocampus of the AAV‐GLS2 + KA group rebounded compared to that in the AAV‐con+KA group, the p62 level was increased and the LC3II level dramatically decreased (Figure 4C). The statistical analyses verified these findings (Figure 4D–G; **p < 0.01, ***p < 0.001, ****p < 0.0001; one‐way ANOVA with *n* = 9 per group). The immunohistochemical results also confirmed the pattern of changes in LC3 in epileptic mice (Figure 4H). The immunofluorescence results indicated that PINK1 and GLS2 were co‐labeled, TOMM20 and GLS2 were also co‐labeled (Figure 4I). Compared with that in the control group, the MFI of PINK1 increased in the AAV‐ con+KA group, but there was no significant difference in the AAV‐GLS2 + KA group; the MFI of GLS2 decreased in the AAV‐con+KA group, but there was no significant difference in the AAV‐GLS2 + KA group. Compared with that in the AAV‐con+KA group, the MFI of PINK1 decreased in the AAV‐GLS2 + KA group; the MFI of GLS2 increased in the AAV‐GLS2 + KA group (Figure 4J,K; **p < 0.01, ***p < 0.001; two‐way ANOVA with *n* = 6 per group).

### 2.5 Overexpression of GLS2 delays the process of mitophagy in epilepsy

**FIGURE 5:**
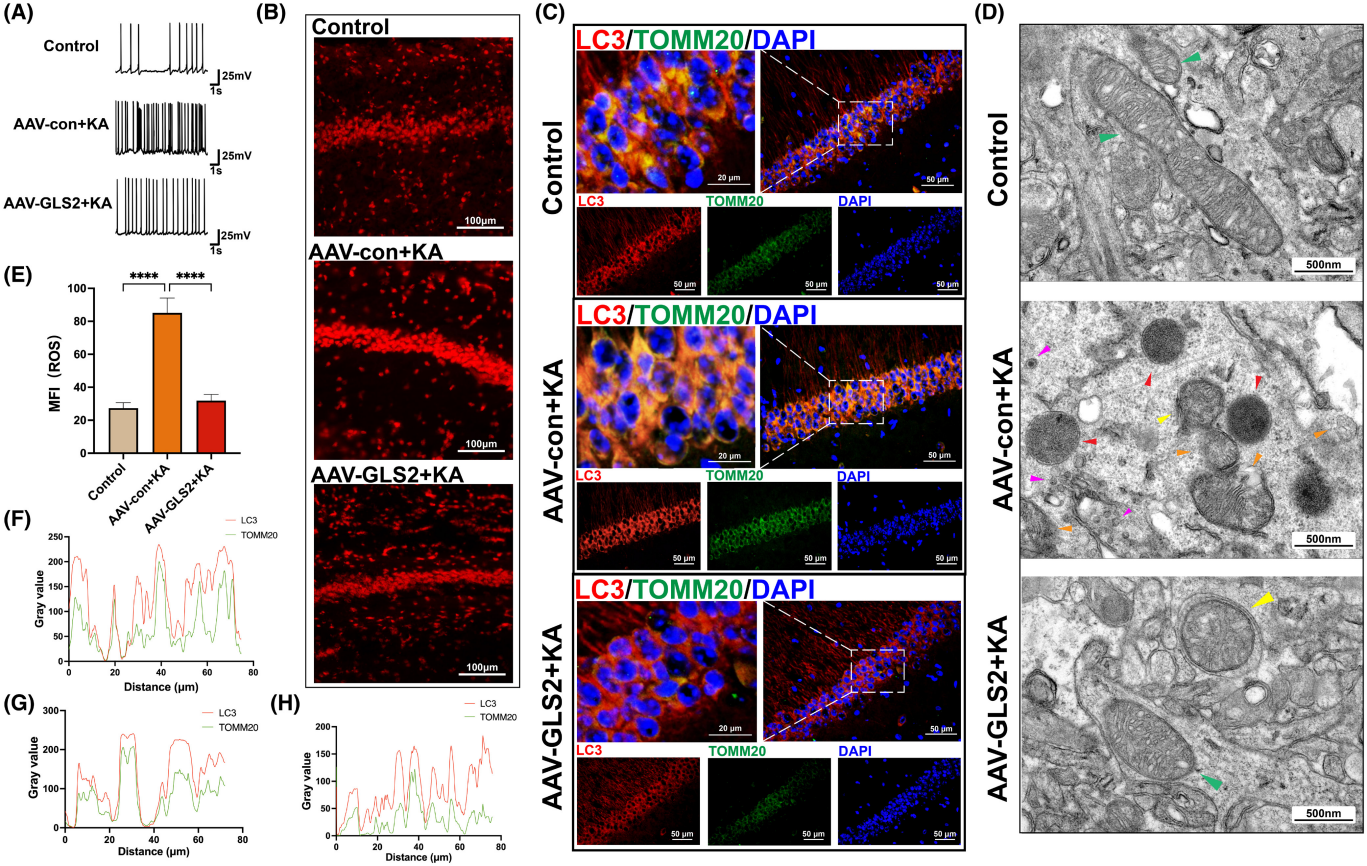
(A) Representative AP images of the hippocampal CA1 region in brain slices. (B) Representative images of ROS in the hippocampal CA1 region (the scale bar = 100 μm) and (E) the corresponding statistical analysis (****p < 0.0001; one‐way ANOVA with *n* = 9 per group). (C) Representative images of immunofluorescence colocalization (the scale bar = 50 μm or 20 μm) of LC3 (red) and TOMM20 (green) and (F–G) the corresponding quantitative analysis results (F, the control group; G, the AAV‐con+KA group; H, the AAV‐GLS2 + KA group). (D) From top to bottom, typical transmission electron microscopy images of hippocampal CA1 neurons in the control group, AAV‐con+KA group, and AAV‐GLS2 + KA group are displayed (the green arrows represent normal mitochondria, the yellow arrows represent spherical mitochondria, the orange arrows represent fragmented mitochondria, the red arrows represent autophagosomes, and the purple arrows represent autophagolysosomes; the scale bar = 500 nm).

First, a whole‐cell patch clamp was used to identify the APs in three sets of brain slices. The findings demonstrated that the AP frequency was lower in AAV‐GLS2 + KA mice than in AAV‐con+KA mice and more frequent in AAV‐con+KA mice than in control mice (Figure 5A). ROS levels in the CA1 region of the hippocampus were measured. Compared with that in the control group, the average fluorescence intensity in the AAV‐con+KA group was significantly greater, while there was no significant difference in the AAV‐GLS2 + KA group (Figure 5B,E). To further confirm the involvement of mitophagy in epilepsy, the colocalization of LC3 (red) and TOMM20 (green) was evaluated to determine the differences in mitophagy levels among the groups. The results showed that compared to that in the control group, the area in which CA1 was colocalized in the AAV‐con+KA group was greatly enlarged.

In contrast, the colocalization area of the AAV GLS2 + KA group was significantly reduced (Figure 5C, F–H). Alterations in neurons in the hippocampal CA1 region of each group of mice were observed via transmission electron microscopy. The control group had intact mitochondrial morphology and membrane structure (indicated by the green arrowhead) without the formation of autophagosomes or autophagolysosomes. In the AAV‐con+KA group, the mitochondria became spherical (indicated by the yellow arrowhead) and damaged, even appeared fragmented (indicated by the orange arrowhead), which was surrounded by autophagosomes (indicated by the red arrowhead) and autophagolysosomes (indicated by the purple arrowhead). The mitochondria of the AAV‐GLS2 + KA group also became spherical and enveloped by a membrane formed by the endoplasmic reticulum. Surrounding autophagosomes formed, but autophagosomes and lysosomes had not yet accumulated in large quantities near mitochondria (Figure 5D).

## 3 DISCUSSION

The present research provides a preliminary analysis of the impact of GLS2 on epileptogenesis and presents three key findings. First, compared to that in the control group, the expression of GLS2 in mice with KA‐induced epilepsy was lower on the seventh day. Second, further studies have shown that overexpression of GLS2 during the latent period of epilepsy reduces susceptibility to chronic epilepsy, reduces epileptic activity according to behavioral tests, and reduces abnormal discharges in LFP recordings. Third, there is an interaction between GLS2 and mitophagy‐related proteins. Specifically, the regulation of GLS2 mainly reverses mitophagy during the latent period of epilepsy by affecting the expression of PINK1, p62, LC3, and TOMM20, thereby reducing the occurrence of SLEs.

Epilepsy is a common neurological disorder caused mainly by abnormal neuronal discharge and membrane potential imbalance. Mitochondrial dysfunction is one of the causes of epilepsy and its development. Mitochondria, important organelles that provide a large amount of energy to neurons, can be engulfed and degraded, leading to abnormal neuronal discharge. The development of epilepsy is closely related to changes in mitophagy function.^22^ PINK1 and LC3 are proteins associated with mitochondrial transport and autophagy processes. Several studies have shown that PINK1 is expressed on mitochondria in the absence of plasmid membrane voltage and can activate LC3‐mediated autophagy. Specifically, PINK1 can promote the initiation of mitochondrial autophagy through the formation of LC3II and mitochondrial immobilization. Therefore, PINK1 and LC3 play crucial roles in regulating mitochondrial autophagy. Mitophagy relies on PINK1 and the ubiquitin ligase Parkin. PINK1 is a mitochondrial kinase that can protect mitochondria from damage and maintain mitochondrial homeostasis. This pathway has been proven to be an essential factor affecting neurological disorders such as Parkinson's disease.^23^ In epilepsy, abnormal mitophagy leads to imbalanced expression of PINK1.^24^ Multiple lines of evidence suggest that the frequency of epileptic seizures is significantly reduced after hippocampal autophagy is improved, which is mainly manifested by the upregulation of LC3 and downregulation of p62.^24^, ^25^, ^26^, ^27^ ROS are reactive oxygen species that are harmful oxidants in cells and can cause oxidative damage to proteins, lipids, and DNA within cells.^28^, ^29^ When excessive production of ROS or impaired intracellular antioxidant capacity occurs, ROS accumulation can occur, leading to mitochondrial dysfunction and cellular damage. In this case, the process of mitochondrial autophagy may be affected, preventing the repair of damaged mitochondria effectively and disrupting the energy balance and physiological function within the cell.^30^, ^31^ GLS2 is an enzyme associated with oxidative stress and is involved primarily in glutamate metabolism and antioxidant responses. GLS2 plays a vital role in regulating ROS levels and cellular oxidative stress. GLS2 can affect the production and clearance of intracellular ROS by regulating glutamate metabolism.^18^, ^32^ In addition, GLS2 can counteract the damage caused by oxidative stress by regulating the synthesis of glutathione (GSH), thereby protecting cells from ROS damage.^33^ These critical relationships are important for maintaining intracellular oxidative balance and normal cellular function. It plays a crucial role in maintaining the steady state of the nervous system.

The initial enzyme involved in glutamine metabolism is GLS, which catalyzes the conversion of glutamine in cells into glutamate. In mammals, phosphate‐activated GLS exists in two varieties: GLS1 (the renal type) and GLS2 (the liver type), which are encoded by distinct genes on distinct chromosomes.^10^, ^11^ GLS2 is a critical regulatory factor for glutamine cleavage and is associated with tumor inhibitory activity.^12^, ^20^ Glutamate is an essential excitatory neurotransmitter in the brain, and dysregulation of glutamate receptors and the glutamatergic system is related to many neurological and psychiatric disorders, especially epilepsy.^34^, ^35^ The main characteristics of epilepsy include recurrent and unpredictable interruptions and abnormal discharges of normal brain function. Most scholars convey that glutamate‐mediated neuronal overexcitation has a pathogenic function in causing epileptic seizures. GLS2 plays a specific role in the oxidative respiratory chain by metabolizing glutamine and glutamate.^16^ Therefore, we conducted a correlation analysis on the concentration of glutamate in the cerebrospinal fluid of epileptic mice (Figure S1). We speculate that the changes in ROS may be caused not only by mitophagy but also by the metabolic mechanisms of glutamine and glutamate in the oxidative respiratory chain. This will also be a direction for our future research.

**FIGURE 6:**
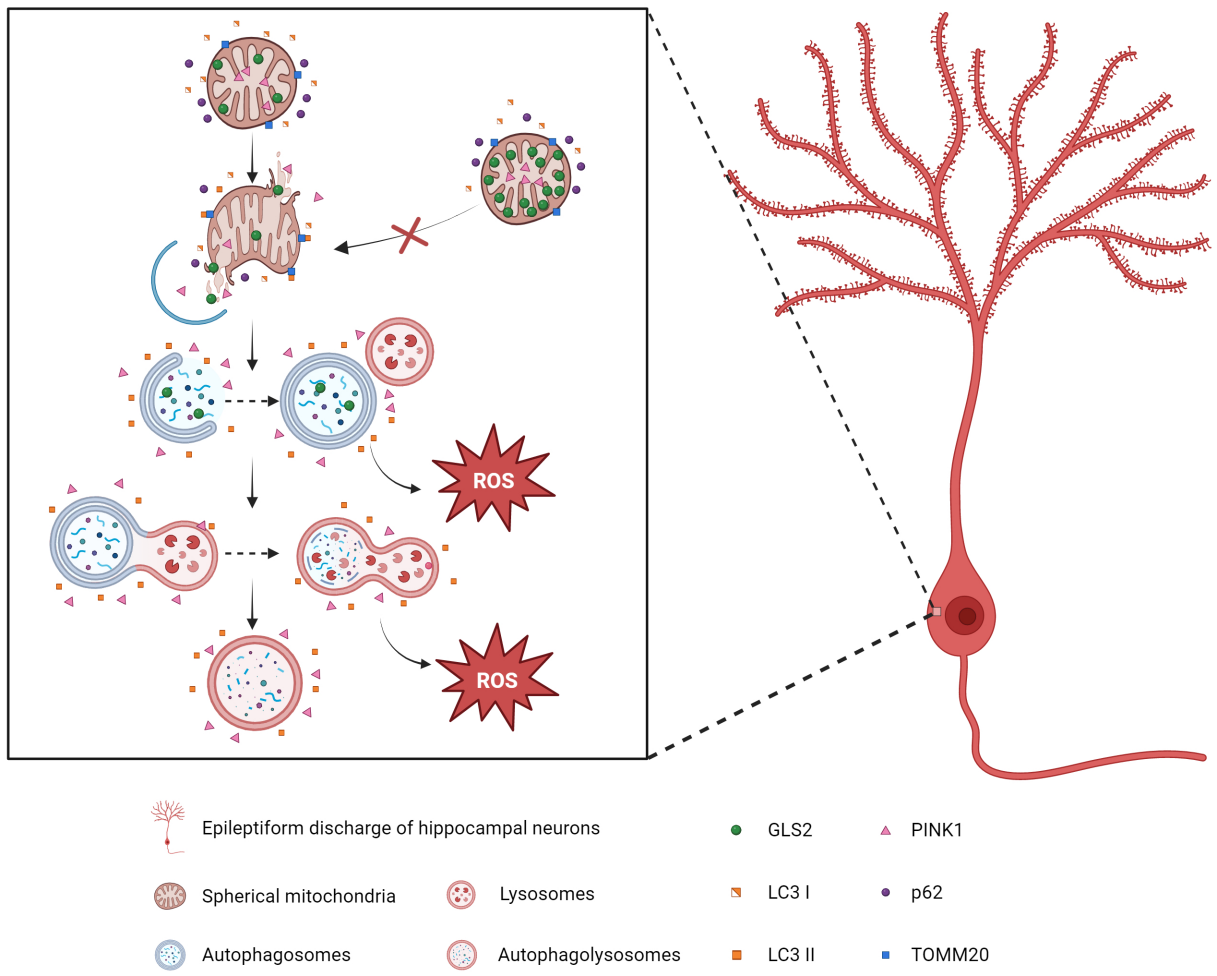
The mechanism diagram of mitochondrial autophagy in epileptic discharge neurons. Overexpression of GLS2 protected the mitochondrial inner membrane from damage and inhibited mitochondrial autophagy in hippocampal neurons with epileptic discharge, thereby reducing the generation of ROS.

Autophagy is activated, as evidenced by an increase in LC3 II and a decrease in p62 protein levels. We hypothesize that GLS2 influences epilepsy by participating in mitophagy processes through the critical role that autophagy plays in epilepsy and its vital role in autophagy. Multiple studies indicate that the process leading to neuronal death involves autophagy‐induced cell death.^36^, ^37^ Here, our results also confirmed these conclusions. Our immunofluorescence double‐labeling results showed that overexpression of GLS2 led to a decrease in the colocalization of TOMM20 and LC3, which indicated that mitophagy was inhibited. According to our transmission electron microscopy results, during the latent period of epilepsy, the mitochondrial structure of hippocampal CA1 neurons in epileptic mice is disrupted, and many autophagosomes gather around damaged mitochondria. The mitochondrial morphology of epileptic mouse neurons overexpressing GLS2 in the hippocampal CA1 area changed, but there was no significant generation of autophagosomes. From our TEM results, it can be seen that mitochondria were enveloped by vesicles, and the formation of these vesicles was precisely the manifestation of early mitophagy. Changes in the shape of the mitochondria typically occur in conjunction with the emergence of these vesicles.^38^, ^39^ These morphological results indicate that mitophagy is delayed by overexpression of GLS2, which inhibits the formation of autophagosomes in hippocampal CA1 neurons during the latent period of epilepsy (Figure 6).

In addition, certain limitations of this study must be noted. First, we did not investigate whether inhibiting the expression of GLS2 accelerates mitophagy. Second, we did not verify whether regulating the level of LC3 affects the expression of GLS2. Third, although GLS2 is a GLS, we did not detect the metabolic levels of glutamine or glutamic acid in epilepsy. These factors are currently unknown and require further research in the future.

In conclusion, we initially discovered that GLS2 is downregulated in the glutamate‐induced neuronal excitatory injury mode, and in the hippocampus in a latent epilepsy mouse model, revealing a hitherto unknown and critical role of GLS2 in epilepsy. Seizures are less likely to occur when GLS2 expression is upregulated in the CA1 area of the hippocampus. Additional research revealed that GLS2 overexpression can inhibit LC3 production and stall the process of mitophagy. Therefore, GLS2 may be a novel marker for predicting future episodes of epileptic activity.

## 4 MATERIALS AND METHODS

### 4.1 Cell culture and treatment

In a 5% CO_2_ incubator at 37°C, immortalized mouse hippocampal neuronal cells (HT22) were cultivated in DMEM supplemented with 10% FBS, 100 units of penicillin, and 100 g/mL streptomycin. The cells were placed in a six‐well plate at a density of 4 × 10^5^ cells well and randomly separated into control and seizure groups, which were treated for 24 h with normal or contained 5 mM glutamate culture medium respectively. The concentrations of the extracellular fluid used were as follows (in mmol/L): NaCl, 145; KCl, 2.5; CaCl_2_, 2; MgCl_2_, 1; HEPES, 10; glucose, 10; and glycine, 0.001.^25^, ^26^ The HT22 cells were then collected for Western blot analysis.

### 4.2 Animals

The Chongqing Medical University Committee on Animal Research authorized all the animal studies performed in this study. All animal experiments followed the National Institutes of Health Guide for the Care and Use of Laboratory Animals and the norms of Chongqing Medical University's Animal Ethical Committee. The ARRIVE 2.0 guidelines were followed for reporting animal data. The Experimental Animal Center of Chongqing Medical University provided healthy adult male C57BL/6 mice (weight range: 16–25 g, age range: 8–10 weeks). All mice were housed in a pathogen‐free (SPF) animal facility with a 12 h/12 h light/dark cycle at a constant temperature (21–22°C) and humidity (50%–60%). Mice had unlimited access to water and food.

### 4.3 KA‐induced epileptic mouse model

Previous research has described the procedures for administering KA intrahippocampally.^40^ Mice were anesthetized with tribromoethyl alcohol (2,2,2‐tribromoethanol) intraperitoneally and placed on a stereotaxic device (RWD Life Science). KA (50 nL of a 20 mM solution; Sigma–Aldrich Co.) was injected into the right hippocampal CA1 region for 3 min using a 0.5 μL syringe (Hamilton). The three‐dimensional positioning coordinates of the CA1 area are anterior–posterior (AP) 2.0 mm, medial‐lateral (MEL) 1.5 mm, and dorsal‐ventral (DV) 1.6 mm. The syringe was left in place for 5 min to reduce reflux before being progressively withdrawn. Mice injected with the same dose of 0.9% physiological saline using the same injection method were defined as the control group.

### 4.4 Construction and injection of adeno‐associated viral vectors

Hanbio Biotechnology produced the adeno‐associated virus (AAV) products utilized in this investigation. The AAV vectors had a titer of 1 × 10^12^ vg/ml. Anesthetized mice were placed on a stereotaxic device. The method for brain stereotactic localization has already been discussed.^41^ AAV vectors expressing the GLS2 coding sequence with three flags at the C‐terminus and green fluorescence (ZsGreen) at the N‐terminus were used to overexpress GLS2; these constructs were subsequently injected into the CA1 region of the mouse hippocampus. These mice were defined as the AAV‐GLS2 group. Furthermore, AAV vectors expressing ZsGreen alone were administered to mice in the AAV‐con group as a negative control. Mice injected with the same dose of 0.9% physiological saline using the same injection method were defined as the control group.

### 4.5 Behavioral assessment

Mice were continually monitored by a video surveillance system (24 h per day) for 1 month after KA injection. SRSs were diagnosed using Racine's scale (stages 0–5).^42^ Only mice in the 4–5 SRSs were included in the epilepsy group. Additionally, hippocampal LFP recordings were obtained to determine the efficacy of the KA‐induced epilepsy mouse model.^43^ The behavioral alterations of the mice in the KA, AAV‐con+KA, and AAV‐GLS2 + KA groups were also examined via video recordings after KA injection, which occurred 3 weeks after AAV vector injection.

### 4.6 Hippocampal LFP recordings

As before, the field potential of the mouse hippocampus was recorded. The LFP density in the mouse hippocampus was measured after behavioral evaluation.^41^, ^44^ A Plexon MAP data collection device was used to record and monitor the LFP, and Neuroexplorer software from Nex Technologies was used for analysis.^45^ Repeated spontaneous clustered waveforms with amplitudes exceeding 1 mV and lasting more than 5 s were defined as seizure‐like episodes (SLEs). The hippocampal LFP density of the AAV‐GLS2, KA, AAV‐con+KA, and AAV‐GLS2 + KA groups of mice was recorded and monitored.

### 4.7 Western blotting

The Western blot procedure has already been previously reported.^40^, ^44^ After the membrane was transferred, the diluted primary antibodies were incubated at 4°C overnight. The primary antibodies used were anti‐GLS2 rabbit polyclonal antibody (1:1000; ZenBioScience), anti‐LC3A/B (D3U4C) XP rabbit monoclonal antibody (1:2000; CST), rabbit polyclonal anti‐SQSTM1/p62 antibody (1:1000; CST), anti‐GAPDH rabbit polyclonal antibody (1:10000; Proteintech), and beta‐actin monoclonal antibody (1:20000; Proteintech). The following day, the proteins on the membranes were visualized by using Hypersensitive ECL Western Blot Substrates (ZenBioScience) on a fusion imaging system (Vilber Lourmat; France) after being incubated for 1 h at room temperature with a goat anti‐rabbit IgG antibody (1:10000; Proteintech) conjugated with horseradish peroxidase. The obtained strip images were analyzed quantitatively using ImageJ 2.9.0 (Media Cybernetics, USA).

### 4.8 Immunofluorescence labeling

The method of making frozen brain slices has been described by previous researchers,^44^, ^46^, ^47^ and after rinsing with PBS, the cell membrane of brain slices was perforated using 0.4% Triton X‐100 at 37°C for 20 min. After three thorough rinses, sodium citrate solution was used to wash the brain slices for 30 min of high‐temperature repair. The mixture was allowed to warm to room temperature and rinsed thoroughly. Then, the slices were blocked with 5% goat serum (Boster) at 37°C for 1 h. The following primary antibodies were subsequently added to the brain slices at the following concentrations and incubated overnight in a 4°C wet box: anti‐GLS2 rabbit polyclonal antibody (1:50; 863,996; Zenbio), anti‐LC3 rabbit monoclonal antibody (1:100; 12,741; CST), anti‐NeuN mouse monoclonal antibody (1:200; MAB377; Sigma), anti‐GFAP rabbit monoclonal antibody (1:400; 250,027; Zenbio), anti‐iba1 mouse monoclonal antibody (1:800; GB12105‐100; Sevicebio) and anti‐TOMM20 mouse monoclonal antibody (1:100; ab283371; Abcam). The next day, the slices were treated in the dark at 37°C for 1 h with a mixture of the following secondary antibodies: CoraLite488‐conjugated goat anti‐mouse IgG (H + L) (1:100; SA00013‐1; Proteintech) and CoraLite594‐conjugated goat anti‐rabbit IgG (H + L) (1:100; SA00013‐4; Proteintech). Finally, the brain slices were stained and sealed with DAPI Fluoromount‐G®(SouthernBiotech). The brain slices were processed according to the above method and imaged by confocal microscopy (Nikon).

### 4.9 Immunohistochemistry

Immunohistochemistry was carried out using previously published methods.^48^ The brain slices were dewaxed in xylene for at least half an h, and the slices were sequentially placed in ethanol solutions from high to low concentrations (100%, 95%, 80%, and 75%) for transparency. The slices were then incubated in 3% H_2_O_2_ at 37°C for 15 min. After being rinsed with PBS, the slides were incubated with goat serum at 37°C for 30 min. The slices were then treated at 4°C overnight with primary rabbit polyclonal anti‐GLS2 antibody (1:50; 863,996; Zenbio), anti‐LC3 rabbit monoclonal antibody (1:100; 12,741; CST), or secondary goat anti‐rabbit antibody (Zhongshan Golden Bridge). The instructions for the DAB staining kit (Zhongshan Golden Bridge) manual were followed for the subsequent experimental steps. Images were obtained using an automated biological microscope (Olympus, Osaka, Japan) after the slides were sealed with neutral resin. All cells with buffy cytoplasm staining were judged to be positive. For the quantitative study of GLS2 and LC3 expression, ImageJ 2.9.0 (Media Cybernetics, USA) was utilized.

### 4.10 Coimmunoprecipitation (Co‐IP) assay

The hippocampus of KA‐induced epileptic mice was placed in centrifuge tubes, and 200 μL of RIPA lysis buffer (Beyotime, P0013D) was added to each hippocampal region to facilitate lysis. The instructions for the protein A/G magnetic beads (MedChem Express, HY‐K0202) were followed for subsequent operations. Then, 30 μL of magnetic beads was rotated and mixed with 2 μL of rabbit monoclonal IgG (CST, 3900), 10 μL of anti‐GLS2 rabbit polyclonal antibody (863,996; Zenbio), 10 μL of anti‐PINK1 antibody (23274‐1‐AP; Proteintech), and 5 μL of anti‐LC3A/B (D3U4C) XP rabbit monoclonal antibody (1:2000; CST), which were incubated for 18 h at 4°C. Next, the antibodies were removed, and PBST was used to clean the magnetic beads and antibody complex thoroughly. The protein lysis solution was added to the magnetic beads and antibody complex overnight at 4°C. The next day, the magnetic bead composites were thoroughly cleaned with PBST, mixed with 1× loading buffer, and denatured in a 95°C metal bath for 15 min.^28^, ^30^ Then, the magnetic beads were discarded, and the denatured samples were collected for Western blot experiments.

### 4.11 Electrophysiological analysis of tissues

Hippocampal slices were generated from the decapitated brains of mice that had received an intraperitoneal injection of tribromoethanol to induce anesthesia. The next step was carried out as previously described.^49^ The pyramidal neurons in the hippocampal CA1 region of the brain slices were recorded by whole‐cell patch clamp using a reversed‐phase contrast microscope (Nikon). Action potentials (APs) at the resting membrane potential were recorded using whole‐cell patch‐clamp recording in current clamp mode.^50^ The current clamp recorded the APs under the resting membrane potential in this area. An internal solution (5 mM NaCl, 90 mM potassium gluconate, 15 mM KCl, 10 mM EGTA, 60 mM HEPES, and 3 mM Na2ATP, pH 7.2) was added to the pipettes (3–5 MΩ polished glass pipettes). The electrophysiological data were collected with a DigiData 1322A (Axon, USA) and MultiClamp 700B amplifier (Axon), and the obtained data were analyzed with pCLAMP 9.2 software (Molecular Devices, USA).

### 4.12 Determination of ROS in brain tissue

A fresh brain tissue sample was embedded in OCT compound to make 16 μm frozen sections on a slicer (Leica, Germany), and the steps in the product manual were followed to determine the ROS in the hippocampal CA1 area using a frozen section tissue ROS assay kit (BestBio Science, Shanghai). Fluorescence images were obtained using laser scanning confocal microscopy (Nikon).

### 4.13 Electron microscopy

The mice were anesthetized with tribromoethanol, and their limbs were restricted. The heart was fully exposed, the needle was inserted through the left ventricle, and the right atrial appendage was cut off; 60 mm of PBS was slowly infused into the mouse body, and then 60 mL of glutaraldehyde was added until the mouse's body stiffened. The skull was opened, and the brain was gently removed. Tissue samples were carefully dissected from the hippocampal CA1 area with a volume of less than 1 mm^3^ and fixed in 2% glutaraldehyde at 4°C overnight; afterward, the samples were incubated at 4°C with 1% osmium for 3 h. The fixative was removed, and the sample was thoroughly rinsed with 0.1 M, pH = 7.0 phosphoric acid buffer. After gradient dehydration, the samples were soaked in alcohol (at five concentrations: 50%, 70%, 80%, 90%, 95%, and 100%) at each concentration for 15 min. Finally, the sample was transitioned to pure acetone for 20 min. The sample was embedded after penetration treatment and heated overnight at 70°C. 70‐nm brain slices were prepared using an ultramicrotome (Leica, Germany) and stained in uranyl acetate‐lead citrate (3%) overnight to obtain the embedded sample. The slices were imaged and imaged by a transmission electron microscope with a pixel resolution of 0.2 nm.^5^, ^6^

### 4.14 Statistical analysis

The Shapiro–Wilk test was used to test for normality in all the data, and the Levene test was used to test for homogeneity of variance. The mean ± standard error of the mean (mean ± SEM) was calculated for all the experiments. For variance, the post hoc Bonferroni method was utilized. A two‐tailed unpaired Student's *t*‐test was used to compare the two groups. For the comparison of three samples, one‐way ANOVA was used. For analysis of variance, two‐factor and three‐sample means were compared, and two‐way ANOVA was used. IBM SPSS 22.0 and GraphPad Prism 9.0 software were utilized for statistical analysis and charting. When *p* < 0.05, the intergroup difference was considered to be statistically significant.

## AUTHOR CONTRIBUTIONS

Yuan Gao, Limin Ma, Jinxian Yuan, and Yangmei Chen designed this research. Yuan Gao, Limin Ma, Yunyi Huang, Yuenan Ban, Peng Zhang, Dandan Tan, and Minxue Liang participated in the experimental operations. Yuan Gao, Limin Ma, Xiaolan Yang, Chen Gong, and Zhipeng Li conducted the data collection. Yuan Gao, Limin Ma, and Yangmei Chen made revisions and edits to this article. All the authors have read and approved this manuscript before submission.

## FUNDING INFORMATION

This research was supported by a grant from the National Natural Science Foundation of China (No. 82071458) and an award from the Chongqing Natural Science Foundation (No. CSTB2022NSCQ‐MSX1203).

## CONFLICT OF INTEREST STATEMENT

All authors declare no conflict of interest.

## Supporting information


Data S1.

## Data Availability

The data that support the findings of this study are available from the corresponding author upon reasonable request.
