# Supplementary material for: GLS2 reduces the occurrence of epilepsy by affecting mitophagy function in mouse hippocampal neurons
Source: CNS Neurosci Ther. 2024 Oct 15;30(10):e70036. doi: 10.1111/cns.70036 (PMC11474837; doi:10.1111/cns.70036)
Supplement: Supplementary file 1 — Data S1. [file CNS-30-e70036-s001.docx]

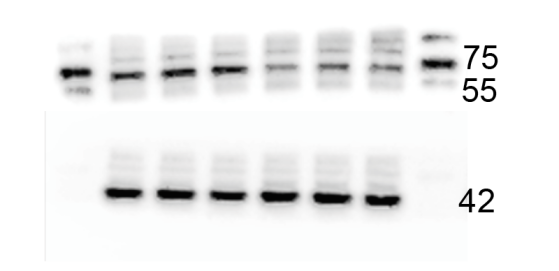
**Figure1 A：**


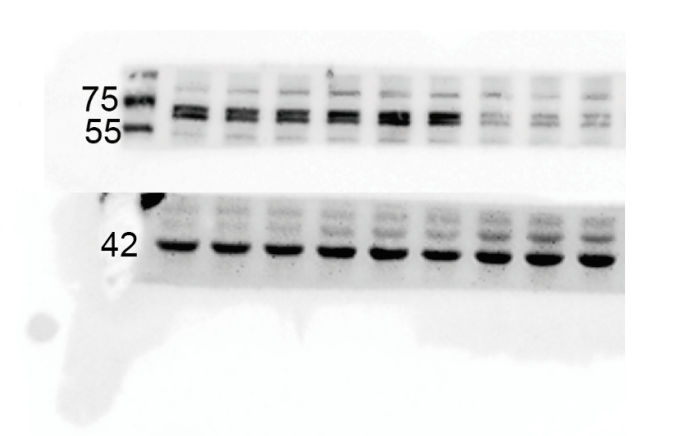


**Figure1 F：**


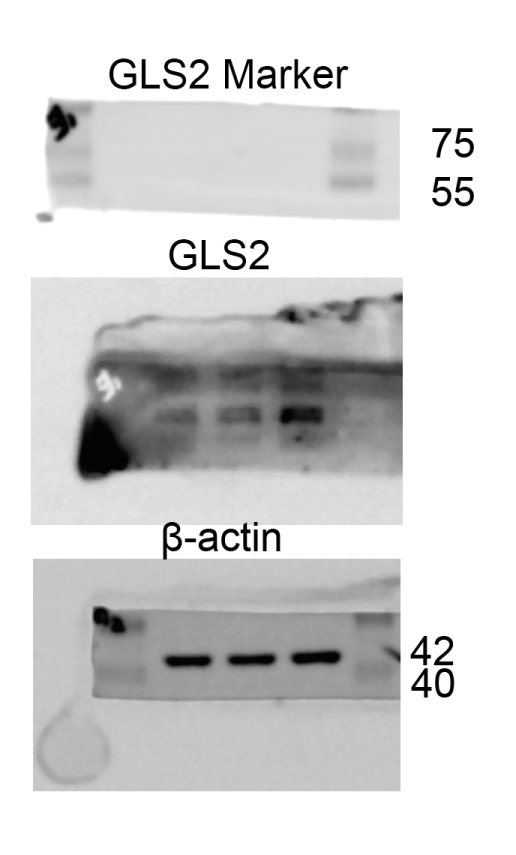


**Figure3 D：**


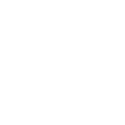


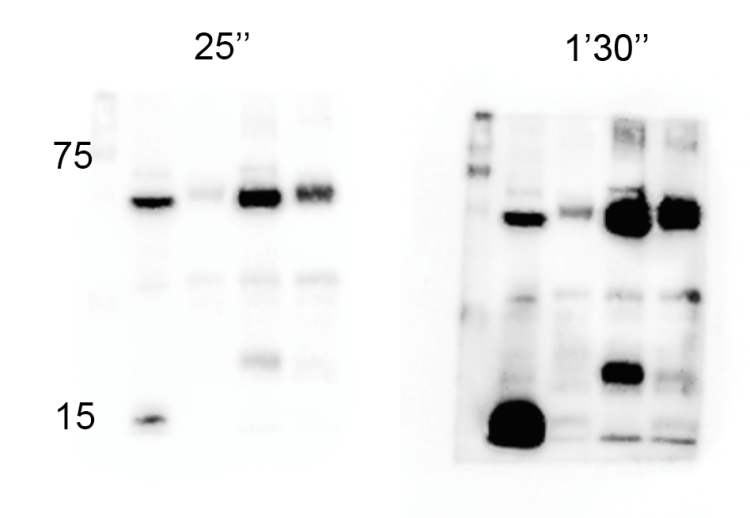


**Figure4 A:**


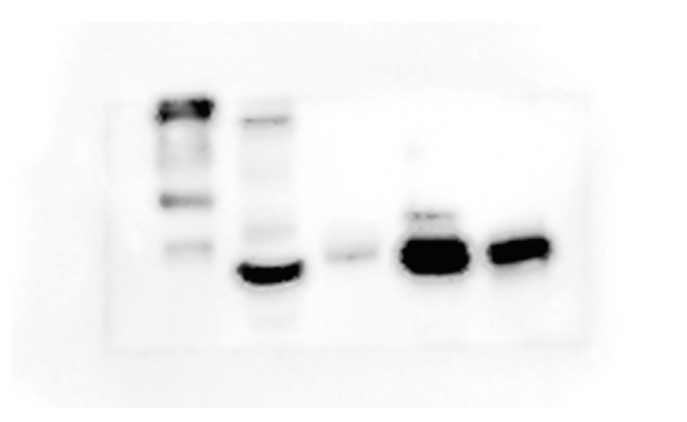


**Figure4 B:**

GLS2


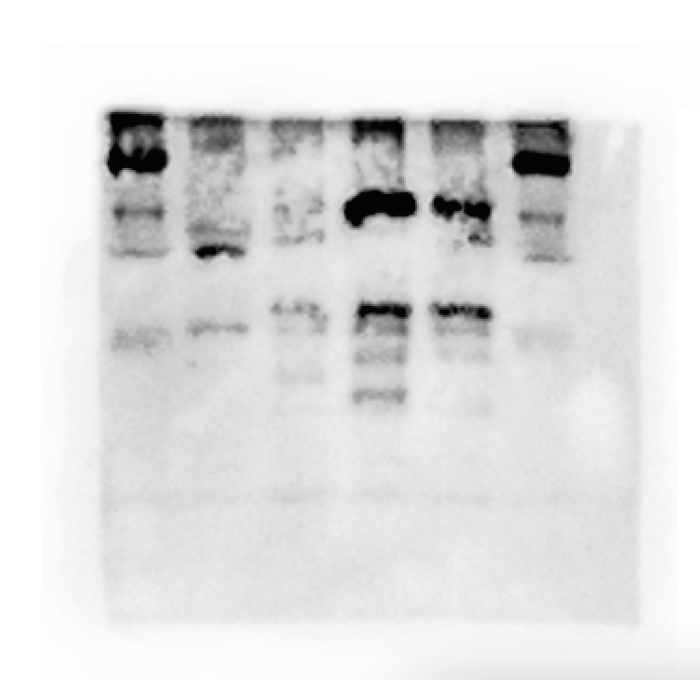


PINK1


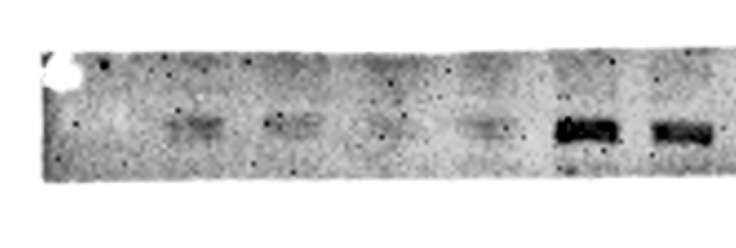
**Figure4 C:**

66


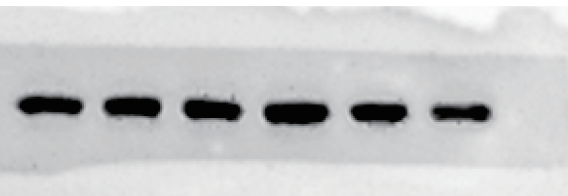


36


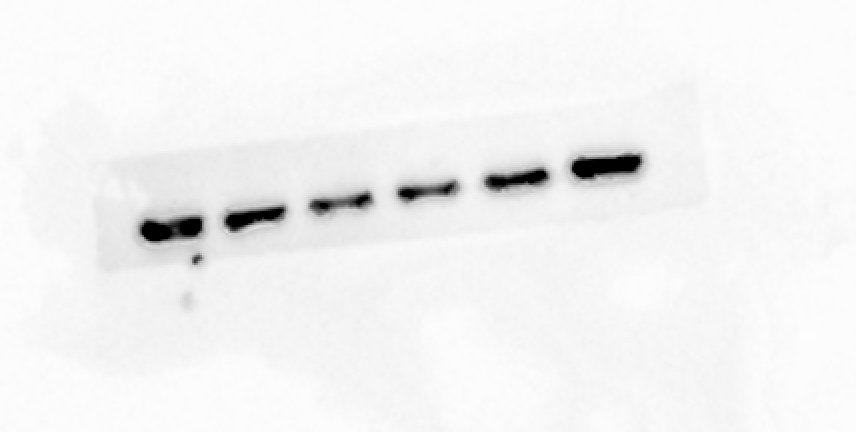


62


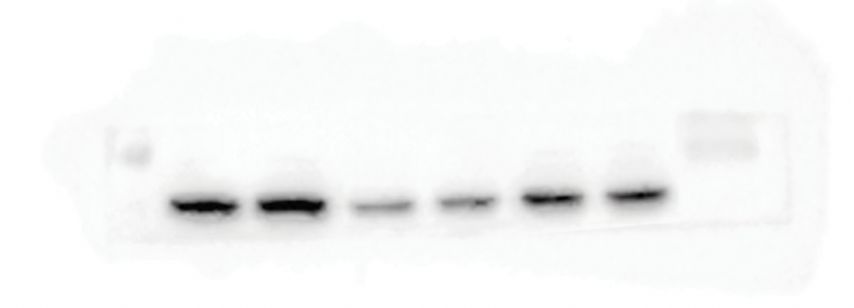


20


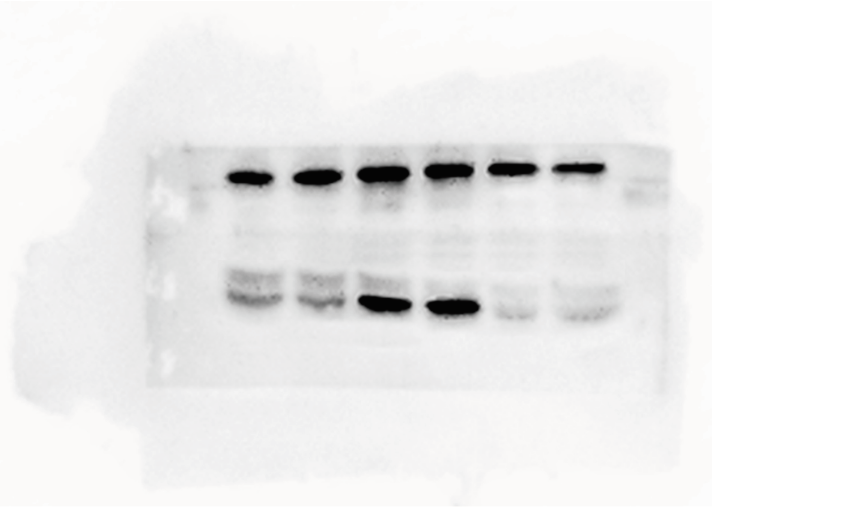


36

15

**Methods:**

**Glutamate concentration measurement**

The amount of glutamate in the cerebrospinal fluid (CSF) was determined using an AmplexTM Red Glutamate/Glutamate Oxidase test kit. To measure the fluorescence intensity, the 96-well plate was put into a multifunctional plate reader. There were two lighting parameters set: 590 nm for the absorbance and 530–560 nm for the excitation. The fluorescence parameter values were used to generate a standard curve, and the sample fluorescence values were used to compute the glutamate concentration.

**Extraction of cerebrospinal fluid**

Following the anesthesia of the C57BL/6J mouse, the head was positioned on a stereotaxic localizer, and a 20 μl microsyringe was inserted. The lateral ventricle's coordinates, which were 1.0 mm posterior and 0.5 mm to the right of the fontanelle, were used to set the syringe location. To aspirate CSF slowly, a 2.5 mm injection depth was utilized, and the syringe was adjusted to negative pressure. From every mouse, ten microliters of translucent, colorless CSF were taken out.

**Results:**

To detect changes in glutamate concentration, we extracted the CSF of mice and measured the glutamate concentration in the cerebrospinal fluid of each group of mice using an AmplexTM Red Glutamate/Glutamate Oxidase test kit. The results of using a multifunctional plate reader showed that compared with the control group, the KA and AAV-con+KA groups increased in glutamate concentration. Compared with the KA group and AAV con+KA group, the AAV-GLS2+KA group showed a decrease in glutamate concentration. There was no significant statistical difference between the control group and the AAV-GLS2+KA group (*P < 0.05, **P < 0.01, ***P < 0.001; Multi-Way ANOVA with n = 6 per group).


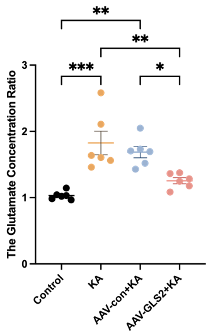


Supplementary Figure 1: Analysis of the CSF glutamate concentration showing that Compared with the control group, KA and AAV-con+KA groups increased the glutamate concentration. Compared with KA and AAV-con+KA groups, AAV-GLS2+KA decreased the glutamate concentration. (*P < 0.05, **P < 0.01, ***P < 0.001; Multi-Way ANOVA with n = 6 per group).
